# Event-related potentials reveal incongruent behavior of autonomous vehicles in the moral machine dilemma

**DOI:** 10.1038/s41598-025-99531-6

**Published:** 2025-05-08

**Authors:** Maren A. K. Bertheau, Cindy Boetzel, Christoph S. Herrmann

**Affiliations:** 1 Experimental Psychology Lab, Department of Psychology, Carl-von-Ossietzky University, Ammerländer Heerstr. 114-118, 26111 Oldenburg, Germany; 2 Department of Informatics, Karlsruhe Institute of Technology (KIT), Am Fasanengarten 5, 76131 Karlsruhe, Germany; 3 European Medical School, Cluster for Excellence “Hearing for All”, Research Center Neurosensory Science, Carl-von-Ossietzky University, Carl-von-Ossietzky-Straße 9-11, 26129 Oldenburg, Germany; 4 Research Center Neurosensory Science, Carl von Ossietzky University, 26129 Oldenburg, Germany

**Keywords:** Autonomous driving, ERP, LPP, Moral machine, P3, trolley problem, Neurophysiology, Psychology, Human behaviour, Neuroscience, Cognitive neuroscience, Social neuroscience

## Abstract

We investigated event-related potentials (ERPs) in the context of autonomous vehicles (AVs)—specifically in ambiguous, morally challenging traffic situations. In our study, participants (n = 34) observed a putative artificial intelligence (AI) making decisions in a dilemma situation involving an AV, expanding on the Moral Machine (MM) experiment. Additionally to the original MM experiment, we incorporated electroencephalography recordings. We were able to replicate most of the behavioral findings of the original MM: In case of an unavoidable traffic accident, participants consistently favored sparing pedestrians over passengers, more characters over fewer characters, and humans over pets. Beyond that, in the ERP we observed an increased P3 (322–422 ms), and late positive potential (LPP) (500–900 MS) amplitude in fronto-central regions when the putative AI’s decision on a moral dilemma was incongruent to the participants’ decision. As P3, and LPP are associated with the processing of stimulus significance, our findings suggest that these ERP components could potentially be used to identify critical, or unacceptable situations during human-AI interactions involving moral decision-making. This might be useful in brain computer interfaces research when, classifying single-trial ERP components, to dynamically adopt an AV’s behavior.

## Introduction

Autonomous vehicles (AVs) may enhance road safety by decreasing accidents, while simultaneously conserving energy and reducing travel times by optimizing traffic flow and avoiding congestion^1^. Additionally, AVs might provide mobility for those who are unable to physically or legally drive, e.g., people with disabilities.

For AVs to achieve widespread adoption, they need to demonstrate acceptable behavior. The rise of personal computer use in offices has demonstrated that new technology needs to be perceived as useful and as easy to use to be widely adopted. In the Technology Acceptance Model (TAM), easy to use means that the technology is clear and understandable, not requiring a lot of mental effort, and being perceived as easy to operate. If the new technology meets both criteria, useful and easy to use, potential users can form an attitude towards using it. Forming an attitude determines if users form a behavioral intention to use said technology which might, according to the TAM, result in actual system use^2^.

So a technology’s behavior has to appear comprehensible to users. For AVs, this can be rather straightforward in strictly regulated traffic situations, e.g., stopping when there is a red traffic signal. However, there are more ambiguous situations in traffic with no clear right or wrong solutions. In these situations, driver decisions may result from factors such as their attitudes, personality, risk aversion, driving experience, etc.^3^. Therefore, it might be advisable to take individual preferences into account and, where possible, adapt or explain the AV’s behavior.

During driving situations, it would be impractical to hand out questionnaires to users to assess whether they perceived the AV’s behavior as comprehensible and subsequently acceptable. Physiological measures may provide an unobtrusive way to assess user perceptions of AV decisions during dynamic and complex driving situations, and subsequently identifying critical, unacceptable situations. Using physiological measures, users would not need to make an active input to the system, by e.g., pressing a button. In perspective, this could have great potential for designing inclusive environments and giving agency back to e.g., users with motor disabilities.

Here electroencephalography (EEG) is a suitable method, as it allows for mobile setups^4^, and has a high temporal resolution, which is necessary to adequately capture responses in dynamic and often times ambiguous traffic situations. A frequently used measure in the EEG is the event-related potential (ERP), a brain response time-locked to the presentation of an event such as the presentation of a stimulus.

As driving situations are complex, requiring higher-order cognitive functions, it is plausible to concentrate on later ERP components: P3 and late positive potential (LPP). Briefly, the P3 is typically elicited by unpredictable, infrequent stimuli^5^. The P3 can be subdivided into two components: the P3a, and the P3b. The P3a appears in frontal regions after unexpected, unusual, surprising, and task-irrelevant stimuli^6–8^. The P3b’s highest peaks are observed in parietal regions after task-relevant^5^, awaited^9^ stimuli. A differentiating feature between P3a and P3b is the component’s location: frontal or parietal, respectively. Further, the P3 is also found to be modulated by the affective content of the stimulus material^10,11^, such as the International Affective Picture system^12^, a standardized set of pictures frequently utilized in emotion research. The LPP is another ERP component associated with higher cognitive processing. It is a sustained positive deflection in the ERP emerging approximately 300 ms after stimulus onset with a maximum at centro-parietal electrodes^13^. It is often elicited by incongruent complex stimulus material such as facial expressions^14^, or affective pictures and words^15^. Further, LPP amplitudes were positively related to self-reports of arousal^16^. From this, and substantial similarities in topography and latency of both components, Hajcak and Foti^13^ theorized that P3 and LPP may be inextricably linked and that they reflect the output of a common neural generator that responds to stimulus significance.

To translate the complex and especially ambiguous situations with AVs to simplified experimental terms, and make it appropriate for ERP research, we deem moral dilemmas particularly well-suited. Like real-life traffic situations, they introduce a certain level of ambiguity while also providing a strong manipulation, which facilitates the detection of specific ERP components. The ambiguity of moral dilemmas of life or death situations in traffic stems from the fact that ethically they do not have a clear solution^17^, evident in a division in ethical philosophy. For instance, consequentialism, and utilitarianism suggest that saving more lives is always the ethical right decision, whereas deontological ethics claim no decision-making at all was ethically defendable^18^. Further, moral dilemmas can be presented in a simplified symbolic manner, as opposed to immersive in field traffic situations, but meanwhile represent a high-stakes situation. This is advantageous when measuring ERPs.

In ERP research, moral dilemmas are examined using a variety of visual stimulus modalities: text^19–24^, static images^25–28^, as well as videos^29,30^.

Sarlo, Lotto, Rumiati & Palomba^23,24^ employed text-based day-to-day variations of the Footbridge dilemma^31^, and text-based day-to-day variations of the Trolley dilemma^32^. The Footbridge dilemma^31^ originally entails sacrificing one individual as an intended means to save others. The Trolley dilemma^32^ originally entails sacrificing one individual as a foreseen but unintended consequence of saving others. Participants showed increased amplitudes in the P260 component of the ERP in frontal and fronto-polar areas when they decided on variations of the footbridge dilemma compared to variations of the trolley dilemma^23,24^.

Fernandes et al.^19^ employed similar stimulus material also entailing text-based variations of Footbridge^31^ and Trolley dilemma^32^, as well as lower conflict situations. They investigated ERP responses to utilitarian (maximizing benefits for the largest number of people) and deontological (avoiding actions that may be intrinsically unacceptable, regardless of the consequences) choices in moral dilemmas across the lifespan. They found that older adults had a higher fronto-central positivity (150–350 ms) than younger adults after utilitarian responses.

Jiang, Zhuo, Wang & Lin^21^ investigated text-based situations based on the Trolley dilemma^32^ where protagonists made binary choices: either the utilitarian choice (taking the action to kill or harm an innocent person but saving more people) or the non-utilitarian choice (taking no action to kill or harm the innocent person but letting several people die). The protagonist was either the participant themselves (“You did ...”) or another person (“Zhang San did ...”). Interestingly, moral judgment for another person’s utilitarian choices elicited larger amplitudes of the frontal LPP (500–900 ms) than when judging their own utilitarian choice.

Gan et al.^20^ have participants judge human agents in elaborate text-based social moral dilemmas. In the later ERP components They find a right side lateralized temporo-parietal effect at 400-500 ms and 580-780 ms (slow wave). Interestingly, when the dilemmas’ agent intentionally caused a harmful outcome, the frontal slow wave (380-780 ms) was less negative than when they were not able to cause harm.

Taken together, these results suggest that text-based moral dilemmas modulate later components (e.g. P260, P3, LPP) in frontal regions^19–24^.

Alternatively, static images depicting diverse social situations between characters are frequently used to investigate ERPs during moral dilemmas^25,26^. Pletti, Decety & Paulus^26^ employ the Chicago moral sensitivity task^33^, a task designed to assess moral judgments in children containing situations such as someone needing help getting back to their feet after falling. In their adult participants, they found a prominently increased LPP (400-1000 ms) at centro-parietal electrodes in antisocial (the protagonist does not help) as compared to prosocial (the protagonist helps) trials. Gui, Gan & Liu^25^ had participants judge moral violations and non-moral scenes from a subset of the International Affective Picture System^12^. For the moral violations Gui, Gan & Liu^25^ found no fronto-parieto-occipital LPP (350–420 ms) effect, but a significantly increased slow wave (450–650 ms) amplitude in moral pictures compared to non-moral ones.

Only Wolff, Gomez-Pilar, Nakao & Northoff^27,28^ utilize a traffic situation in the broadest sense. Specifically, they employed a simplified version of the footbridge dilemma, represented by two groups of stick figures and instructed participants to make moral judgments. Wolff, Gomez-Pilar, Nakao & Northoff^27,28^ observed a less positive P300, and more positive late LPP (1,000–2,000 ms) at Pz for morally challenging dilemmas compared to those with less ambiguous resolutions. Taken together, image-based moral dilemmas produce mixed effects on the P3 window as well as the LPPs (400–1000 ms) with a broad scalp distribution^25–28^.

Video-based moral dilemmas are less frequently investigated using EEG^29,30^. When participants make moral judgments about prosocial (interpersonal assistance) and antisocial (interpersonal harm) actions in presented videos, Yoder & Decety^30^ find differences in the LPP amplitudes. Similarly, when human agents intentionally vs. accidentally cause harm in videos depicting social dilemmas, Cenka, Spaccasassi, Petkovic, Pezzetta, Arcara & Avenanti^29^ find effects in later central and posterior ERP components ($\sim 400$ ms).

Summarizing the previously discussed ERP results, despite the use of a wide variety of stimulus material, many studies report effects in later components such as the P3 and the LPP at frontal and parietal sites. This is in line with Hajcak & Foti^13^’s theory, that P3 and LPP may reflect output from a common system that tracks the time-course of stimulus significance. This suggests that later components are associated with the higher level cognitive processing essential for a mental assessment of moral dilemmas^34^. Thus, P3 and LPP could be informative to identify unacceptable ambiguous situations, when investigating moral dilemmas in the context of AVs. Additionally, across all stimulus modalities, previous studies focused on how participants themselves^19,23–28^ or human agents^20,21,29,30^ made moral decisions. None of the previously employed moral dilemma stimulus material shed light on participants’ ERP when an artificial agent, e.g., an AV, is making a (moral) decision. The present investigation aims at this gap in existing research.

A suitable stimulus material to investigate this is present in Awad et al.^35^’s famous and thorough Moral Machine (MM) experiment. Behaviorally, they investigated moral dilemmas in the context of AVs. Participants’ behavior towards this stimulus material is well documented. In the original MM task, participants were presented with variations of the trolley dilemma^36^. These scenarios depicted unavoidable traffic accidents with two possible outcomes, determined by whether the AV swerved or stayed on course. Participants were instructed to choose their preferred outcome (see “Methods” for an example). The MM task is well suited for ERP studies as it consists of static traffic image vignettes with ambiguous solutions, which allows for controlled yet ecologically valid experiments. To our knowledge, the MM has not yet been utilized for such purposes.

In the present study, we aimed to investigate the extent to which ERPs can be used to capture participants’ acceptance of a putative AI’s decisions in ambiguous traffic situations simulated in a laboratory environment. Therefore, we used a modified version of Awad et al.^35^’s MM experiment. In our experiment, participants first made a decision based on an image-based vignette (see “Methods” ). Subsequently, they were shown the outcome of a hypothetical AI’s decision, which could either align with (congruent) or differ from (incongruent) their own choice. The value of this approach lies especially in connecting theoretical and practical aims. It broadens the basic neurophysiological understanding of social moral dilemmas towards human-AI interaction. Additionally, this is especially interesting for application: In perspective, our ERP results could contribute to realize online evaluation of human-AI interaction bypassing obtrusive techniques such as questionnaires.

To establish plausibility of our participants’ responses to the chosen stimulus material we aimed to replicate Awad et al.^35^’s two largest and two smallest effects. Note that we did not pursue an exhaustive replication of Awad et al.^35^. We expected participants to display:

- *Intervention*: a preference in favor of inaction - hence having the AV remain on its lane;
- *Relation to AV*: a preference in favor of sacrificing the passengers in the AV rather than pedestrians;
- *No. of Characters*: a preference in favor of sparing more characters;
- *Species*: a preference in favor of sparing humans rather than pets; and furthermore
- *Acceptability*: we expected that participants would rate incongruent trials as less acceptable than congruent ones.

Based on Based on Hajcak & Foti^13^’s suggestion that increased P3 and LPP amplitudes are associated with stimulus significance, we hypothesized the presentation of the putative AI’s decision in incongruent trials (see “Methods” ) to increase the P3 and LPP amplitude contrast to congruent trials.

## Results

### Behavioral results

**Fig. 1:**
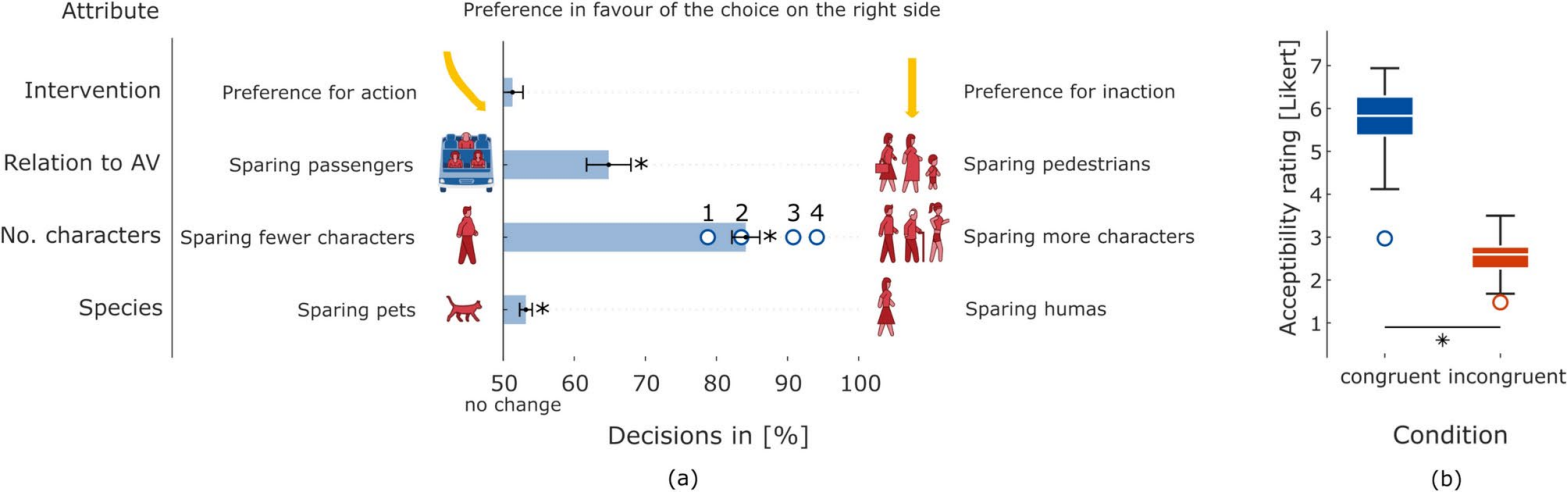
**(a)** Participants’ decisions after dilemma presentation (see “Methods” ) for the four dichotomous attributes *Intervention*, *Relation to AV*, *No. of Characters*, and *Species*. Each row gives the portion of preference in favor of the choice on the right side from the total amount of decisions made on this attribute in percent (%). Whiskers indicate standard error (SE)s. Significant effects $(p<.05)$ are marked with ’*’. If the proportion between choices was 50%, there would be no preference for either choice. The higher the percentage, the higher the preference for the choice on the right side. For the attribute ’No. of characters’ additional circles indicate the preference for each number of additional characters spared separately. The characters presented here are exemplary. The full set of possible characters can be found in Awad et al.^35^ or supplementary Fig. 1. **(b)** Acceptability ratings of the putative AI’s decision (see “Methods” ). The Likert scale ratings with ’completely acceptable’ (7) and ’completely unacceptable’ (1) are shown separately for both conditions congruent (blue) and incongruent (red). On each box, the central mark indicates the median, and the bottom and top edges of the box indicate the 25th and 75th percentiles, respectively. Outliers are plotted individually as circles.

Overall, participants were more likely to remain in the same lane, than intervening by swerving onto the other lane (see Fig. 1a). However, this difference was not statistically significant (a) *Intervention*: $t_{(33)}=0.934, p=0.357, d=.160$). Participants showed a preference in favor of sacrificing the AV’s passengers over pedestrians (b) *Relation to AV*: $t_{(33)}=4.761, p<.001, d=.861$), a preference in favor of saving more characters in comparison to less (c) *No. of Characters*: $t_{(33)}=18.134, p<.001, d=3.110$), and favor for sparing humans over pets (d) *Species*: $t_{(33)}=3.665, p<.001, d=.629$). Overall, participants behaved in line with the findings from Awad et al.^35^.

Furthermore, participants rated the acceptability of the AI higher when its decision was congruent with their own decision (e) *Acceptability*: $t_{(33)}=19.095, p<.001, d=3.275$) (see Fig. 1b and supplementary Table 1 for further details).

### EEG results

In the following, the results of the two analyses of variance (ANOVAs) (*Condition x Electrodes*) for the components P3, and LPP are presented.

#### P3 results

**Fig. 2:**
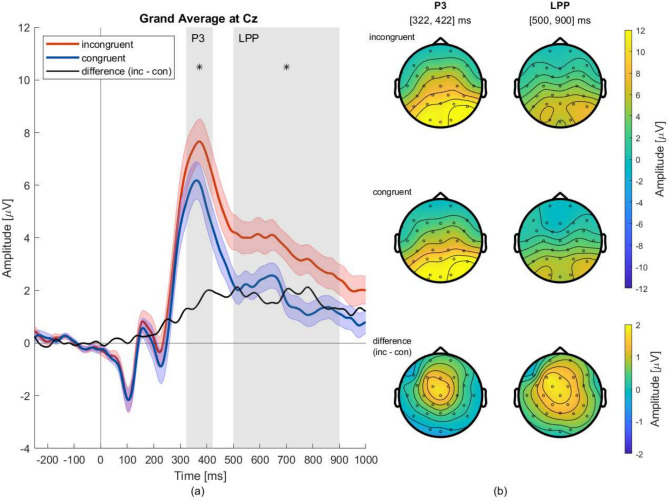
**(a)** shows the baselined (– 200 to 0 ms) ERPs timelocked to the presentation of the AI’s decision (see “Methods” ). For both conditions, incongruent (red), congruent (blue), as well as their difference (solid black) are shown at electrode Cz. The shaded error regions indicate the SE. The gray shading indicates the time intervals corresponding to the topographies. * indicate significant time intervals. **(b)** The topographies show the ERP components P3 and LPP column wise in chronological order. The first row shows topographies from the incongruent, and the second row from the congruent condition. The third shows their difference. Note that the two conditions share one color bar, whereas we used a smaller color bar for the difference between conditions.

For the P3, we found a significant main effect of *Condition* on the amplitude ($\mu$V) ($F_{(1,33)}=9.887$, $p=.004$, $eta ^2=.231$) (see supplementary Table 2). During the P3 (322–422 ms) the amplitude was greater in incongruent vs. congruent trials (see Fig. 2a).

**Fig. 3:**
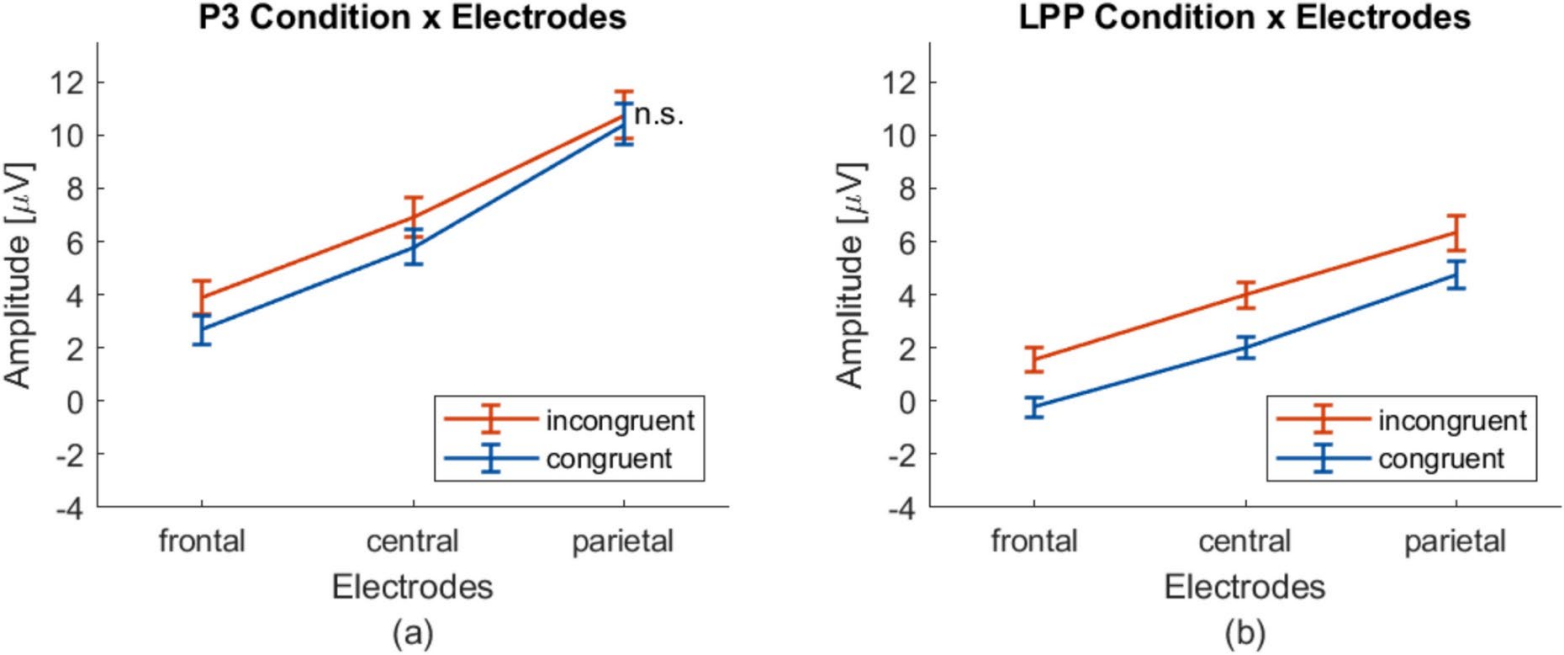
**(a)** The significant interaction* Condition x Electrodes* at the component P3 from the overarching ANOVA (see supplementary Table 2). All visible differences were significant in post hoc procedures. Only the difference inc-con in parietal electrode P3 marked with ’n.s.’ was not (see supplementary Table 5.) **(b)** Note, that the interaction *Condition x Electrodes* was not significant at component LPP (see supplementary Table 3). Whiskers indicate SEs.

There was also a significant main effect on *Electrodes* ($F_{(2,66)}=98.464$, $p_{(Greenhouse-Geisser(GG))}<.001$, $\eta ^2=.749$) (see supplementary Table 2). Post-hoc t-tests ($C = 12$ relevant comparisons) revealed that all differences between sites were found to be significant: fronto-central $t_{(33)}=-8.883$, $p<.001$, $d=-1.523$; fronto-parietal $t_{(33)}=-10.494$, $p<.001$, $d=-1.800$; centro-parietal $t_{(33)}=-9.116$, $p<.001$, $d=-1.563$ (see supplementary Table 4). This becomes clearer in the context of Fig. 2b. The amplitude of the P3 descriptively was the highest in the occipital sites, which consistently declines with further anterior sites. The interaction plots underpin this finding (see Fig. 3a): in both conditions, the highest amplitude is found on parietal sites, and then in descending order on central and frontal sites.

Furthermore, we found a significant two-way interaction *Condition x Electrodes* ($F_{(2,66)}=18.641$, $p_{(GG)}<.001$, $\eta ^2=.361$) in the P3 time window (see supplementary Table 2 and Fig. 3a). Post-hoc t-tests ($C = 12$ comparisons) showed eight of nine differences significant (range of the t values and Cohen’s ds: $t_{(33)}= |3.661 - 11.768|$, $p<=.010$, $d = |.628 - 2.018|$; see supplementary Table 5). Only the difference incongruent - congruent at the parietal electrodes was significant (see supplementary Table 5). This becomes clearer in the context of Fig. 2b. For the P3 time window, the topography plot shows the difference between congruent and incongruent trials (third row) was located most prominently in frontal and central electrodes whereas it declines towards parietal sites (see Fig. 3a): The difference between conditions is significant in frontal and central electrodes but not at parietal sites.

#### LPP results

For the LPP, we found a significant main effect of *Condition* on amplitude ($\mu$V) ($F_{(1,33)}=24.658$, $p<.001$, $\eta ^2=.428$) (see supplementary Table 3). Similar to the P3, the LPP shows a descriptive increase in the amplitude in incongruent trials as compared to congruent trials (see Fig. 2a).

There was also a significant main effect of *Electrodes* ($F_{(2,66)}=80.168$, $p_{(Greenhouse-Geisser(GG))}<.001$, $\eta ^2=.708$) (see supplementary Table 3). Post-hoc t-tests ($C = 3$ comparisons) revealed that all differences between sites were significant: fronto-central $t_{(33)}=-9.761$, $p<.001$, $d=-1.674$; fronto-parietal $t_{(33)}=-9.322$, $p<.001$, $d=-1.599$; centro-parietal $t_{(33)}=-7.519$, $p<.001$, $d=-1.290$ (see supplementary Table 6). Similar to the P3, for the LPP in both conditions descriptively the topographies show the highest amplitudes in occipital sites which consistently declines with further anterior sites (see Fig. 3b).

The interaction *Condition x Electrodes* was not significant$(F_{(2,66)}=1.609, p_{({GG})}=.215)$ (see Fig. 3b, and supplementary Table 3). For the LPP time window in the topography plot showing the difference between congruent and incongruent trials (see Fig. 2b, third row) the activity was distributed on larger scalp regions as compared to at P3. In contrast to the P3, the difference between the LPP amplitudes of the congruent and the incongruent ERP is also significant at parietal sites (see Fig. 3b).

.

## Discussion

The present study’s main aim was to investigate the extent to which ERPs can be used to capture participants’ acceptance of an AV’s decisions in ambiguous traffic situations simulated in a laboratory environment. The core findings are an increased P3 (322–422 ms), and late positive potential (LPP) (500 to 900 ms) amplitude in fronto-central regions when the AV’s decision on a moral dilemma was incongruent to the participants’ judgment.

First, we briefly address the behavioral analysis, whose primary aim was to assess the plausibility of participants’ responses, rather than to replicate the findings of Awad et al.^35^ exhaustively. Consequently, we focused on replicating a subset of their effects using simplified analyses, which was overall successful: In line with our hypothesis and findings from Awad et al.^35^, participants favored sparing pedestrians over passengers, more characters over fewer characters, and humans over pets. Descriptively, there was an inclination favoring inaction (see Fig. 1a), however, contrary to Awad et al.^35^’s findings, this effect was not statistically significant. Additionally, as expected, participants rated congruent trials as significantly more acceptable than incongruent trials (see Fig. 1b).

Differences in experimental design between Awad et al.^35^’s and our study might help explain why participants did not significantly favor inaction (*Intervention*) in our study. Our laboratory study involved a notably reduced sample size, comprising 34 participants, as compared to Awad et al.^35^ in their online study which had a sample size of over 492,921 participants. Furthermore, Awad et al.^35^’s study drew from a comprehensive database of 40 million decisions, while our dataset was limited to 6,800 decisions. This disparity in sample size and dataset magnitude likely had a pronounced effect on our study’s statistical power, and statistical precision and sensitivity, which renders the detection of subtle effects challenging. Additionally, it is worth noting that in the original study, favoring inaction was the smallest effect among their reported effects. The difference in experimental settings, such as being in a private environment versus a laboratory setting, may have also influenced participants’ behavior towards favoring action. Furthermore, to achieve comparability between participants we adhered to a fixed set of vignettes. It is possible that this choice inadvertently resulted in the formation of a vignette subset that favored action-oriented decisions, potentially skewing the overall outcomes in favor of action over inaction.

To summarize the behavioural findings, participants mostly behaved as we would expect based on the results of Awad et al.^35^, which leads us to infer that our participants engaged thoroughly with the task.

Therefore, we can more thoroughly discuss the ERP results: For the P3 and the LPP, there was a significant main effect *Condition*. Based on qualitative observation of the grand-average waveforms, this difference in amplitude between congruent and incongruent trials (Fig. 2a, black line) was minimal during the early ERP components. It then progressively increased during the P3 time window. It reached its peak shortly after the P3, during the LPP, and began to gradually decline around 1,000 ms after stimulus onset.

During the time interval of the P3, there was a significant interaction *Condition x Anterority*. Single main effects revealed that the differences between the conditions were most prominent in fronto-central, but not in parietal regions (see Fig. 3a). This pattern was also reflected in the differences’ topography (Fig. 2b, third row): The difference in amplitude emerged fronto-centrally during the time interval of the P3 and spread during the LPP into parietal regions. The frontal distribution of the differences in activity and the rather early latency of the P3 peak (372 ms) may suggest that our stimulus material elicited a P3a. A P3b instead would have been located more posteriorly as compared to the anteriorly distributed P3a^6–8^. This suggests that participants perceived the AI’s incongruent decisions as surprising^6–8^. Given that both P3 and LPP were observed in both conditions, the significant effect of *Condition* (congruent vs. incongruent) following the AI’s decision reflects a modulation of these components.

Subsequently, during the LPP (500–900 ms) the observed difference attains its maximum magnitude. An explanation for that might be that LPPs are often associated with stimulus significance^13,37^ especially related to rather complex stimuli (e.g., pictures, or words) requiring higher-order cognitive functions to be appropriately processed. Hajcak & Foti^13^ define the significance of a stimulus as the extent to which a stimulus activates appetitive or aversive motivational systems. Given that our stimulus material involved moral dilemmas concerning life-or-death situations, it seems plausible that participants found incongruent trials—where the AI’s decisions to sacrifice characters conflicted with their own judgments—particularly significant.

When comparing our results to existing empirical studies on the effect of moral dilemmas on specific EEG components^21,27,28^ we find similarities:

Consistent with our findings, Wolff, Gomez-Pilar, Nakao & Northoff^27,28^ observed a P3 effect at Pz when comparing difficult to easily solvable moral dilemmas as well as an increased late LPP. We also found an increased P3 (322–422 ms). Additionally, we found an increased LPP (500–900 ms), which occurred earlier than the late LPP (1000 - 2000 ms) observed by Wolff, Gomez-Pilar, Nakao & Northoff^27,28^. In contrast to the present study, Wolff, Gomez-Pilar, Nakao & Northoff^27^ and Wolff, Yao, Gomez-Pilar, Shoaran, Jiang & Northoff^28^ found a centro-parietal effect, while our effect was located more fronto-centrally. It has to be noted that in the studies by Wolff, Gomez-Pilar, Nakao & Northoff^27,28^, participants made moral judgments, whereas, in the present paradigm, participants were additionally confronted with an AI’s decision. Hence, tasks were not completely equivalent, which might explain the difference in results. Jiang, Zhuo, Wang & Lin^21^ observed a diminished amplitude of the frontal LPP (500–900 ms) when moral judgments were made on behalf of the participants themselves as opposed to others. For these conditions, they reported no differences in the amplitudes of earlier components, which is descriptively in line with our findings. Thus, our finding of frontal LPP aligns with previous research. However, in our incongruent condition an AI made the decision whereas in^21^’s design, a third person resolved the dilemma. Furthermore, we compared congruent and incongruent decisions made by an AI, rather than contrasting decisions made by the self with those made by an AI. Another difference between Jiang, Zhuo, Wang & Lin^21^’s and the present study, is that they used a text-based moral dilemma task. Hence, performing their task required reading a text, which involves different processing steps as performing the present image-based task. Therefore, even though related, our findings stem from majorly different experimental procedures, but elicit comparable ERP responses which suggests the LPP is a rather stable effect across different experimental setups and contexts in line with^13^, and^37^. Based on this, we argue that when users monitor an AI, the presence of an increased P3 and LPP could serve as an unobtrusive indicator for significant or unacceptable situations in the context of autonomous driving.

While this study provides valuable insights into human-AI interaction, there are limitations to consider before further interpretation.

In the present study, we investigated static image dilemma vignettes, which are especially suited for ERP analysis. However, the dilemma vignettes could be considered as extreme and unlikely situations. To bring our findings closer to real-world traffic applications, it would be beneficial— though challenging—to investigate the effects of AI decisions in a more ecologically valid setting, such as with dynamic stimulus material. The challenges for ERP research would primarily involve ensuring accurate timing, to enable distinguishing between different ERP components which usually lie within only $\sim 100$ ms distances. In dilemma literature, moving imagery such as videos depicting social interactions^29,30^ or vignettes akin to shooter games^38^ have been studied, however, to our knowledge more realistic daily traffic situations have not been covered.

Further, the source of the LPP may not be solely cortical but also involve deeper structures, such as the limbic system and the amygdala, which are not easily recordable through EEG. The use of neuroimaging methods such as magnetencephalogramm (MEG) or functional magnetic resonance imaging (fMRI) might be useful to assess higher-order cognitive processing in the context of moral decision-making as these methods are suitable to map deep brain structures. However, fMRI exhibits a lower time resolution which makes it not suitable to measure an evoked potential. Further, both mentioned imaging methods are expensive and expansive and therefore not well suited for in-field application.

In the field of brain computer interfaces (BCI), there is a vast field that employs single trial classifiers to identify specific ERP components such as error potentials, for adopting an AI’s behavior^39,40^. In previous studies, the classifiers have been trained with data from situations providing clear correct and incorrect outcomes. As our findings revealed differences in the amplitudes of later components (P3 and LPP) for more ambiguous situations, it may be beneficial to include these components when training classifiers. This could enable the unobtrusive evaluation of reciprocal communication between humans and autonomous machines such as AVs during road use.

In the future, the present findings could potentially be used to enable real-time evaluation of situations by AI based on physiological parameters while driving. Initial attempts to implement real-time analysis of physiological parameters while driving have already been made^41,42^. To successfully and reliably archive this, further research is needed to enhance the signal-to-noise ratio in mobile EEG setups.

To conclude, when observing an artificial agent, such as an AV, making moral decisions incongruent with participants’ judgments compared to congruent decisions, we found increased amplitudes of the P3 and a LPP in fronto-central regions. As the P3 and LPP are associated with stimulus significance^13,37^, our findings suggest that human-AI interactions during moral decision-making seem to involve higher-order cognitive processes. Further research is needed to allow the findings to be incorporated into single trial classifiers which might bring current findings from basic research closer to application.

## Methods

### Participants

Thirty-six volunteers were recruited to participate in this study to achieve sufficient statistical power in our design. Participants were included in the study if they were over 18 years old, right-handed, native German speakers, had normal or corrected to normal vision, and had no psychiatric or neurological diseases. Furthermore, they were required to have a valid German driver’s license and be eligible to drive under German law (see §31 StVZO and §316 StGB) at the time of the experiment. All participants gave written informed consent and received 10€ per hour for participation. The research protocols were approved by the Commission for Research Impact Assessment and Ethics (“Komission für Forschungsfolgenabschätzung und Ethik”) at the Carl von Ossietzky University of Oldenburg and complied with all relevant ethical regulations. Two participants were excluded because they participated in only one of two recording sessions. Therefore, 34 participants’ data (mean age = 24.471, SD age = 2.997, age range = 19-34 years, female = 16) were included in the analysis.

### Procedure

The experiment consisted of two sessions, conducted on different days (days between sessions: mean = 6.618, SD = 8.707). During each session, participants were asked to perform the MM task and a Left Turn (LT) task. One session took $\sim 150-200$ min. The order of the tasks was counterbalanced across sessions and participants. The current paper discusses the data from the MM task. The first experimental session began with participants filling in the informed consent and demographic questionnaires. On both days, the EEG, and the peripheral physiological sensors, were positioned. Participants were then seated in an electrically shielded, dimly lit chamber at $\sim 120$ cm distance in front of a display. After a short training to get participants acquainted with the tasks consisting of three (LT task) or six (MM task) congruent trials, the main experiment began. The LT task was divided into four $\sim 20$ min blocks. The MM task was divided into two $\sim 10$ min blocks. Between the blocks, participants had the opportunity to take short, self-paced breaks. At the end of the second experimental day, they were debriefed that they did not interact with an AI but a deterministic abstract machine. Table 1 summarizes the overall procedure.

**Table 1:** Overview on procedure.

Task	Day I				Day II			
	LT test run	LT	MM test run	MM	MM test run	MM	LT test run	LT
Conditions	con	con & inc	con	con & inc	con	con & inc	con	con & inc
Trials	3	4 × 25	6	2 × 50	6	2 × 50	3	4 × 25
Time [min]	$\sim$3	4 × $\sim$20	$\sim$1	2 × $\sim$10	$\sim$1	2 × $\sim$10	$\sim$3	4 × $\sim$20

### Moral machine task

With kind permission from Awad et al.^35^ we used their original stimulus material from the MM experiment. We also followed the wording of the instructions of their original study as closely as possible.

**Fig. 4:**
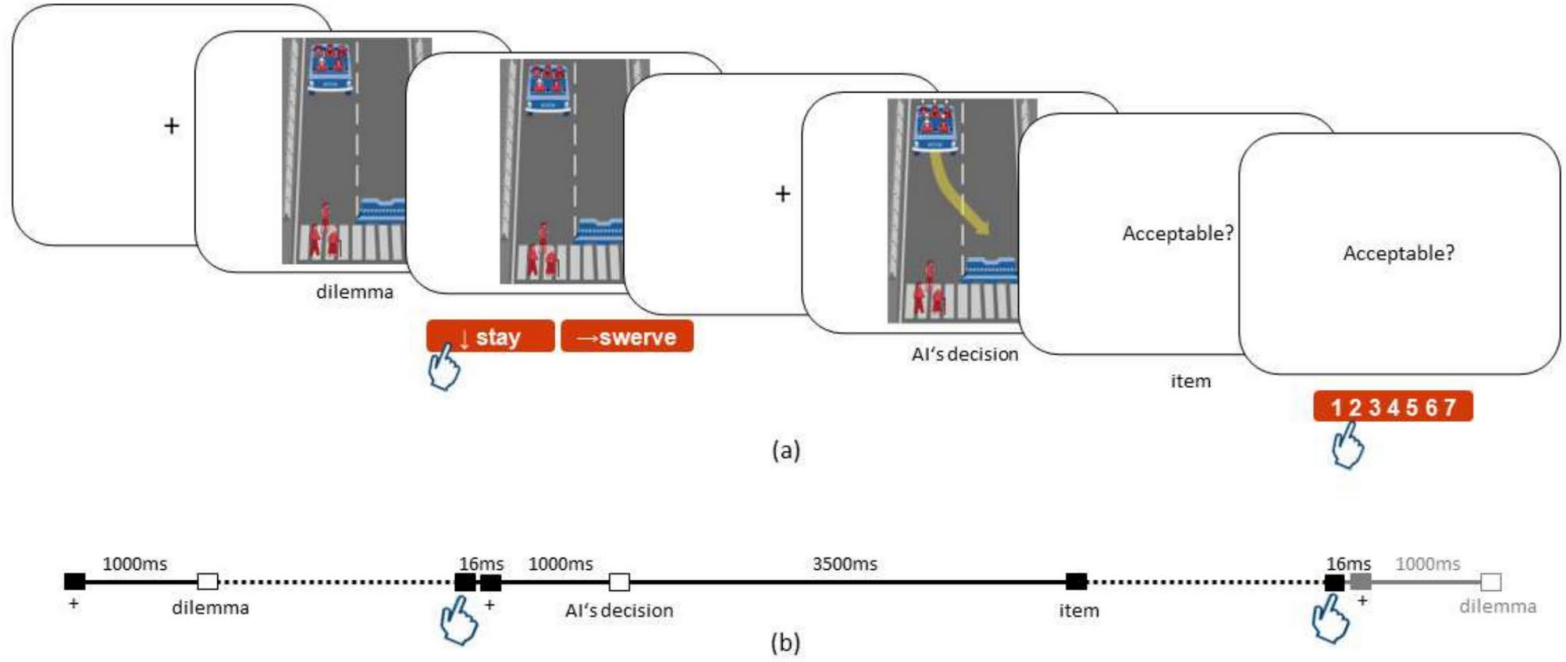
**(a)** Schematic overview of an exemplary incongruent trial of the MM task. The trial consists of fixation crosses ’+’, the presentation of the ’dilemma’, in this example either sacrificing five passengers or three pedestrians, the participant’s decision (1st cursor), the putative AI’s decision, the acceptability item, and the participant’s Likert rating (1–7) (2nd cursor). The items’ exact wording is found in the supplementary material. **(b)** The timeline where solid lines indicate fixed presentation times in milliseconds and dotted lines indicate windows where participants could freely take their time. The filled squares on the timeline correspond to the onset of events from (**a**). White squares symbolize an activation of the photo sensor field (for more information see supplementary material).

Each trial began with a fixation cross that was presented for 1,000 ms. Then, participants were shown an image vignette of a moral dilemma (Fig. 4). The vignettes showed unavoidable accident vignettes with two possible outcomes, depending on whether the AV swerved or stayed on course. Then, participants were asked to decide which outcome they preferred—e.g., sparing the three pedestrians on the crosswalk or the five passengers in the AV (see Fig. 4). Participants were instructed to ’answer based on their gut instinct’ when they were ready and to indicate their opinion using the left and right arrow buttons on a standard keyboard. After they pressed an arrow, a fixation cross was presented for 1,000 ms followed by an image vignette showing the putative AIs decision (henceforth referred to as *the AI’s decision* (see Fig. 4). The AI’s decision was indicated with a yellow arrow and skulls floating over the sacrificed characters’ heads. The decision was displayed for 3,500 ms, as pilot tests suggested that this was sufficient for participants to process the AI’s decision vignette. The AI’s decision could be either congruent or incongruent with the participants’ decision. After each trial, participants were asked to indicate how acceptable they found the AI’s decision on a Likert scale ranging from 1 (completely unacceptable) to 7 (completely acceptable). The chosen range provides the advantage of a neutral middle category (4) and differences are still distinguishable in the human working memory^43^.

We created 100 different vignettes. The vignettes varied in three elements: type, location, and number of character(s). Overall, there were 20 red characters differing in sex (male, female), age (baby, child, adult, elderly), species (human, pet), fitness (large, fit), and social status (homeless, executive) (see supplementary Fig. 1 for the full set of characters). In each vignette, one to five characters pseudo-randomly appeared in two of three possible locations: in the av as passengers, or on either the left or the right side of the crosswalk as pedestrians. When passengers were present, the side of the roadway without passengers was blocked by a wall (see Fig. 4).

In the original study by Awad et al.^35^, there was also a condition of (un-)lawful behavior with traffic lights either being red or green. However, we omitted this condition in our study as our pilot testing showed that the traffic lights were small and easily overlooked by participants. Furthermore, since we were looking for ERP data, this could have led to jittering latencies, because we could not assume that participants were always consciously aware of the traffic lights.

Each participant was presented these vignettes twice in random order—once with a congruent and once with an incongruent AI decision. Thus, each participant decided on a total of 200 vignettes.

### Apparatus

#### Hardware

The stimulus material was presented on a Samsung SyncMaster display: P247GH, 1920 × 1080 pixels, 60 Hertz (Hz) refresh rate.

The EEG data were recorded using a 32-channel actiCAP snap electrode system with the standard 10-10 layout (BrainProducts GmbH, Gilching, Germany). The reference electrode was placed on the tip of the nose and a vertical electro ecculogramm (EOG) electrode was placed under the right eye. The ground electrode was placed at electrode location FPz. Thus, the final EEG signal was acquired from 30 active Ag-AgCl Electrodes. Impedances were measured prior to recording and kept below 20 k$\Omega$. Signals were digitized at a rate of 1000 Hz by a 16-bit ActiChamp amplifier.

#### Software

The task was programmed in Psychtoolbox^44–46^, a toolbox run in MATLAB 2021a (The MathWorks, Inc., Natick, MA, USA). Physiological signals were recorded using BrainVision Pycorder software (BrainProducts GmbH, Gilching, Germany). Data preprocessing and plotting was performed using the FieldTrip toolbox (^47^; http://fieldtriptoolbox.org) in MATLAB 2022b (The MathWorks, Inc., Natick, MA, USA). All statistical analyses were computed using SPSS 29 (IBM, Armonk, NY, USA).

### Data analysis

#### EEG preprocessing

We updated the EEG triggers using photo sensor data to correct the lag between the stimulus onset and the EEG trigger onset, and hence ensure our timing (see supplementary material for details). Trials with missing keyboard entries from the participants were excluded. The EEG data were then demeaned, detrended, and filtered from 1 to 35 Hz. Then, the data were cut in 1,000 ms epochs without overlap. Any epochs containing data exceeding a threshold of 1,000 $\mu$V were rejected. Then, an ICA was performed and components containing eye and muscle movement artifacts were identified visually and rejected. Then, ICA components were projected back onto the raw EEG data. To calculate ERP, raw EEG data were filtered from 0.1 to 20 Hz. Data were then segmented into 4,500 ms epochs, 1,500 ms before each trigger and 3,000 ms after each trigger. Trials with a $$ z \ge \ $$15 using accumulated z-score thresholding (ft_artifact_zvalue) were rejected^47^. Trials from the two experimental sessions were merged and averaged over the trigger at *AI’s decision* (see Fig. 4). Then, we applied a baseline correction from – 200 to 0 ms so that the average of the pre-stimulus period was set to 0 and calculated a grand average ERP over participants.

From the grand average we extracted the ERP components ’P3’, and ’LPP’. For the ’P3’, we defined a search window from 250 to 1,000 MS, where we identified the maximum amplitude ($\mu$V) on the electrode ‘Pz’^48^. The timepoint of the maximum amplitude ± 50 ms was defined as the time window for the analysis of the P3. Since the LPP often does not have clear peaks, we defined a window from 500 to 900 ms (see gray shading in Fig. 2a). This window was chosen to avoid overlap with the P3 window and is typical of previous time windows chosen for LPP analysis, e.g.,^11,21,26,27,49^. The amplitudes in the two components windows were then averaged and exported for inference statistics for the two conditions (congruent and incongruent), and the nine electrodes (F3, Fz, F4, C3, Cz, C4, P3, Pz, and P4). To increase the signal-to-noise ratio for further analysis we aggregated channels to ’frontal’ (F3, Fz, F4), ‘central’ (C3, Cz, C4), and ‘parietal’ (P3, Pz, P4). The electrode selection was based on the most frequently used electrodes in previous literature investigating moral dilemmas in the EEG^21,23–27,30,38,49,50^.

#### Statistical analysis

For the behavioral data, we performed repeated measures t-tests on:

- *Intervention*: the number of left arrow presses versus the number of right arrow presses;
- *Relation to AV*: the number of AVs with passengers sacrificed versus the number of AVs with passengers spared;
- *No. of Characters*: the mean number of characters spared versus the mean number of characters sacrificed;
- *Species*: the number of pets sacrificed versus the number of pets spared, and
- *Acceptability*: the acceptability ratings in the congruent versus the incongruent condition.

On the exported ERP we compute two two-way repeated measures ANOVAs with the factors *Condition* (congruent, incongruent) × *Electrodes* (frontal, central, parietal) for both relevant components P3, and LPP. For the three effects from this repeated measures design with 34 complete datasets, and a conservatively assumed correlation between repeated measures of $r =.2$, we achieved a strong statistical power of $1-\beta \ge .95$ for a conventionally medium sized effect $(f =.25)$^51–53^ (see supplementary Methods for details). To account for the sphericity problem GG corrections were used to correct the degrees of freedom (df). Furthermore, as post-hoc procedure to further disentangle significant three-level main effects and interaction effects, we employed Bonferroni corrected t-tests for *C* relevant comparisons on the simple main effects.

## Supplementary Information

Supplementary Information.

## Data Availability

The data are available from the corresponding author upon reasonable request.
