# Supplementary material for: Event-related potentials reveal incongruent behavior of autonomous vehicles in the moral machine dilemma
Source: Sci Rep. 2025 May 8;15:16048. doi: 10.1038/s41598-025-99531-6 (PMC12062281; doi:10.1038/s41598-025-99531-6)
Supplement: Supplementary file 1 — Supplementary Information. [file 41598_2025_99531_MOESM1_ESM.pdf]

Supplementary Material to "Event-Related Potentials Reveal Incongruent Behavior of Autonomous Vehicles in the Moral Machine Dilemma"

Maren A. K. Bertheau[1,2], Cindy Boetzel[1], and Christoph S. Herrmann[1,3,4,*]

[1]Department of Psychology, Experimental Psychology Lab, Carl-von-Ossietzky University, Ammerländer Heerstr. 114-118, 26111 Oldenburg, Germany
[2]Department of Informatics, Karlsruhe Institute of Technology (KIT), Am Fasanengarten 5, 76131 Karlsruhe, Germany
[3]European Medical School, Cluster for Excellence “Hearing for All”, Research Center Neurosensory Science, Carl-von-Ossietzky University, Carl-von-Ossietzky-Straße 9-11, 26129 Oldenburg, Germany
[4]Research Center Neurosensory Science, Carl von Ossietzky University, 26129 Oldenburg, Germany
[*]christoph.herrmann@uni-oldenburg.de

ABSTRACT

This supplementary material contains further details on Results and Methods for the study "Event-related potentials (ERPs) reveal incongruent behavior of autonomous vehicles (AVs) in the Moral Machine (MM) dilemma".
Regarding Results we supplement the complete tables on the behavioral t-tests (see Table 1), the two two-way analysis of variancess (ANOVAs) for the components ’P3’ (see Table 2), and ’late positive potential (LPP)’ (see Table 3), as well as the corresponding post hoc procedures for those effects which showed to be significant in the ANOVAs (see Tables 4-6).
Regarding Methods we give a complete Character Atlas from the MM stimulus material (see Fig. 1), further details on technical equipment, the used question items, and the statistical power estimation.

Results

			Mean		Difference between means					Significance		Effect
Dependent variable	A	B	A	B	A-B	standard deviation (SD)	standard error (SE)	degrees of freedom (df)	T	two-sided p	p*(C=4)	Cohen’s d
No. arrows pressed	Inaction (left) -	Action (right)	102.650	97.150	5.500	34.353	5.892	33	.934	.357	>1	.160
No. AVs with passengers	Sacrificed -	Spared	86.820	47.059	39.765	48.705	8.353	33	4.761	**<.001**	**<.001**	**.816**
Mean No. of characters	Spared -	Sacrificed	3.590	2.409	1.180	.380	.065	33	18.134	**<.001**	**<.001**	**3.110**
Species: pets	Sacrificed -	Spared	41.471	36.471	5.000	7.954	1.364	33	3.665	**<.001**	**.003**	**.629**
Acceptibility (Likert [1,7])	congruent -	incongruent	5.744	2.541	3.203	.978	.168	33	19.095	**<.001**		**3.275**

Supplementary Table 1 behavioral t-Test results from the MM task with Bonferroni correction with C=4 relevant comparisons.

factor	sphericity correction	square sum	df	MSS	F	p	partial η^2
Condition	-	40.418	1	40.418	9.887	**.004**	**.231**
error(Condition)	-	134.903	33	4.088			
Electrodes	-	1828.809	2	914.404	98.464	<.001	.749
	Greenhouse-Geisser (GG)	1828.809	1.220	1498.724	98.464	**<.001**	**.749**
error(Electrodes)	-	612.920	66	9.287			
	GG	612.920	40.268	15.221			
Condition * Electrodes	-	7.228	2	3.614	18.641	<.001	.361
	GG	7.228	1.454	4.971	18.641	**<.001**	**.361**
error(Condition*Electrodes)	-	12.796	66	.194			
	GG	12.796	47.986	.267			

Supplementary Table 2 Two-way ANOVA for P3 component with GG correction.

factor	sphericity correction	square sum	df	MSS	F	p	partial η^2
Condition	-	161.623	1	161.623	24.658	**<.001**	**.428**
error(Condition)	-	216.300	33	6.555			
Electrodes	-	809.371	2	404.686	80.168	<.001	.708
	GG	809.371	1.135	713.043	80.168	**<.001**	**.708**
error(Electrodes)	-	333.166	66	5.048			
	GG	333.166	37.458	8.894			
Condition * Electrodes	-	1.522	2	.761	1.609	.208	.046
	GG	1.522	1.210	1.257	1.609	.215	.046
error(Condition*Electrodes)	-	31.217	66	.473			
	GG	31.217	39.945	.782			

Supplementary Table 3 Two-way ANOVA for LPP component with GG correction.

simple main effect		diff. between means	SD	SE	95% confidence interval for difference		T	df	significance			effect
					lower bound	upper bound			one sided p	two sided p	p*(C=12)	Cohen's d
frontal -	central	-3.072	2.017	.346	-3.776	-2.368	-8.883	33	<.001	<.001	**<.001**	**-1.523**
frontal -	parietal	-7.303	4.058	.696	-8.719	-5.888	-10.494	33	<.001	<.001	**<.001**	**-1.800**
central -	parietal	-4.231	2.707	.464	-5.176	-3.287	-9.116	33	<.001	<.001	**<.001**	**-1.563**

Supplementary Table 4 Bonferroni corrected t-tests with C= 12 relevant comparisons as post hoc procedure for the three level main effect Electrodes at the component P3.

factor	simple main effect		diff. between means	SD	SE	95% confidence interval for difference		T	df	significance			effect
						lower bound	upper bound			one sided p	two sided p	p*(C=12)	Cohen's d
frontal	(inc -	con)	1.187	1.485	.255	.669	1.705	4.663	33	<.001	<.001	**.001**	**.800**
central	(inc -	con)	1.124	1.791	.307	.499	1.749	3.661	33	<.001	.001	**.010**	**.628**
parietal	(inc -	con)	.359	1.882	.323	-.297	1.016	1.113	33	.137	.274	3.287	.191
con	(frontal -	central)	-3.104	1.881	.323	-3.760	-2.447	-9.620	33	<.001	<.001	**<.001**	**-1.650**
con	(frontal -	parietal)	-7.718	3.824	.656	-9.052	-6.383	-11.768	33	<.001	<.001	**<.001**	**-2.018**
con	(central -	parietal)	-4.614	2.614	.448	-5.526	-3.702	-10.292	33	<.001	<.001	**<.001**	**-1.765**
inc	(frontal -	central)	-3.040	2.212	.379	-3.812	-2.269	-8.015	33	<.001	<.001	**<.001**	**-1.375**
inc	(frontal -	parietal)	-6.889	4.351	.746	-8.408	-5.371	-9.232	33	<.001	<.001	**<.001**	**-1.583**
inc	(central -	parietal)	-3.849	2.839	.487	-4.839	-2.858	-7.905	33	<.001	<.001	**<.001**	**-1.356**

Supplementary Table 5 Bonferroni corrected t-tests with C= 12 relevant comparisons as post hoc procedure for the interaction effect Electrodes x Condition at the component P3.

simple main effect		diff. between means	SD	SE	95% confidence interval for difference		T	df	significance			effect
					lower bound	upper bound			one sided p	two sided p	p*(C=3)	Cohen's d
frontal -	central	-2.333	1.393	.239	-2.819	-1.846	-9.761	33	<.001	<.001	**<.001**	**-1.674**
frontal -	parietal	-4.878	3.051	.523	-5.942	-3.813	-9.322	33	<.001	<.001	**<.001**	**-1.599**
central -	parietal	-2.545	1.973	.338	-3.233	-1.856	-7.519	33	<.001	<.001	**<.001**	**-1.290**

Supplementary Table 6 Bonferroni corrected t-tests with C= 3 relevant comparisons as post hoc procedure for the three level main effect Electrodes at the component LPP.

Methods

Photo Diode Field

We used a photo sensor (Brain Products Gilchingen, Germany) field in the lower left corner of the screen to acquire a ground truth verification of our set up's timing: Simultaneously to the onset of our stimuli, their appearance on the screen, the photo sensor field flipped from grey to white for 100 ms. By comparing these time stamps the to the electroencephalography

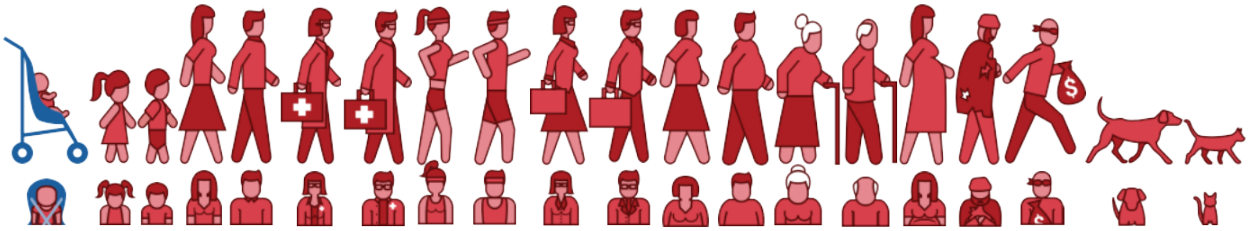

Supplementary Fig. 1 Complete set of 20 characters as pedestrians (top) and passengers (bottom) in the MM task. They differ in sex (female, male), age (baby, child, grown up, elderly), species (human, pet), fitness level (large, fit), and social status (homeless, executive).

(EEG)-triggers we archived an even more precise timing.

Exact Wording of Acceptability Item

German: 'Wie akzeptabel fanden Sie das Verhalten des selbst-fahrenden Fahrzeugs?

völlig unakzeptabel 1 - 2 - 3 - 4 - 5 - 6 - 7 völlig akzeptabel'

English: 'How acceptable did you find the behavior of the self-driving vehicle?

completely unacceptable 1 - 2 - 3 - 4 - 5 - 6 - 7 completely acceptable'

Statistical Power Estimation

G*power Version 3 [1, 2] does not allow for power estimations of ANOVAs with repeated measures on two factors [3]. To evade this we adjusted the effect sizes f according to the method suggested by Rasch, Friese, Hofmann & Naumann [3] (Chapter 3.3). For two-factor ANOVAs, they suggest adjusting f based on the other factor: $f' = \sqrt{q} * f$ or $f' = \sqrt{p} * f$, respectively. Statistical power $(1-\beta)$ estimations were carried out for all three effects of the 2x3 ANOVA *Condition x Electrodes* with 34 complete datasets, and a conservatively assumed correlation between repeated measures of $r = .2$. We employed the effect conventions suggested by G*Power: small $(f = 0.10)$, medium $(f = 0.25)$, and large $(f = 0.40)$. For the main effect *Condition* (congruent, incongruent: p = 2) we employed the following adjustment $f' = \sqrt{q} * f$ to G*Power and found:

- small: $f' = \sqrt{3} * 0.1 = 0.173, \lambda = 2.550, 1-\beta = 0.341$
- medium: $f' = \sqrt{3} * 0.25 = 0.433, \lambda = 15.937, 1-\beta = 0.972$
- large: $f' = \sqrt{3} * 0.4 = 0.693, \lambda = 40.798, 1-\beta = 0.999$

For the main effect *Electrodes* (frontal, central, parietal: q = 3) and the interaction *Condition x Electrodes* we employed the following adjustment $f' = \sqrt{p} * f$ to G*Power and found:

- small: $f' = \sqrt{2} * 0.1 = 0.141, \lambda = 2.549, 1-\beta = 0.267$
- medium: $f' = \sqrt{2} * 0.25 = 0.354, \lambda = 15.942, 1-\beta = 0.947$
- large: $f' = \sqrt{2} * 0.4 = 0.566, \lambda = 40.802, 1-\beta = 0.999$

Thus, overall we at least achieved statistical power of $1-\beta = 0.947$ for the three ANOVA effects assuming a conventionally medium sized effect.

References

1. Faul, F., Erdfelder, E., Lang, A.-G. & Buchner, A. G*Power 3: A flexible statistical power analysis program for the social, behavioral, and biomedical sciences. *Behavior Research Methods* **39,** 175–191 (2007).
2. Faul, F., Erdfelder, E., Buchner, A. & Lang, A.-G. Statistical power analyses using G*Power 3.1: Tests for correlation and regression analyses. *Behavior Research Methods* **41,** 1149–1160 (2009).
3. Rasch, B., Friese, M., Hofmann, W. & Naumann, E. *Quantitative Methoden. Band 2* 5th ed. Supplementary material retrieved from `https://www.lehrbuch-psychologie.springernature.com/sites/default/files/2022-01/Rasch_A5_978-3-662-63283-3_Kapitel_3_GPower_R_Ergaenzung.pdf` (Springer, Heidelberg, 2021).
